# Exploiting the pathway structure of metabolism to reveal high-order epistasis

**DOI:** 10.1186/1752-0509-2-40

**Published:** 2008-04-30

**Authors:** Marcin Imielinski, Calin Belta

**Affiliations:** 1 Center for Applied Genomics, Children's Hospital of Philadelphia, Philadelphia, USA; 2 MD/PhD Program, University of Pennsylvania School of Medicine, Philadelphia, USA; 3 Bioinformatics Graduate Program, Boston University, Brookline, USA

## Abstract

**Background:**

Biological robustness results from redundant pathways that achieve an essential objective, e.g. the production of biomass. As a consequence, the biological roles of many genes can only be revealed through multiple knockouts that identify a *set *of genes as essential for a given function. The identification of such "epistatic" essential relationships between network components is critical for the understanding and eventual manipulation of robust systems-level phenotypes.

**Results:**

We introduce and apply a network-based approach for genome-scale metabolic knockout design. We apply this method to uncover over 11,000 minimal knockouts for biomass production in an *in silico *genome-scale model of *E. coli*. A large majority of these "essential sets" contain 5 or more reactions, and thus represent complex epistatic relationships between components of the *E. coli *metabolic network.

**Conclusion:**

The complex minimal biomass knockouts discovered with our approach illuminate robust essential systems-level roles for reactions in the *E. coli *metabolic network. Unlike previous approaches, our method yields results regarding high-order epistatic relationships and is applicable at the genome-scale.

## Background

The complexity of biological systems arises from the highly parallel and epistatic relationships between their components. Determination of the systems level role of biological components has been classically approached through the study and characterization of knockout phenotypes. However, the robustness of biological systems obscures the role of most individual components, which are redundant and seemingly "dispensible". The role of such robust modules can only be revealed through multiple knockout of complex *essential sets *[1,2].

This property of complex biological systems is particularly true for small-molecule metabolism, which is governed by an intricate and highly robust network of biochemical reactions that leaves most single knockouts without a discernible *in vivo *or *in silico *phenotype. A recent study by Deutscher et al. employs *in silico *multiple knockout analysis to reveal novel essential roles for reactions in the yeast metabolic network [1]. These results indicate the presence of "deep" epistatic relationships between large sets of network components that manifest only with multiple knockouts. They suggest that systematic probing of such complex "essential reaction sets" can establish novel links between reactions and systems level functions.

Interest in epistasis between biological network components is motivated by practical considerations. Firstly, the emergence of microbial resistance motivates the search for new antibiotics. Systems biology can inform this search through "rational" identification of multivalent pharmaceutical targets via a functional analysis of biological network robustness. Secondly, higher order interactions detected in *in silico *network models can help suggest "epistatic" candidate gene combinations for genomic association studies linking genotype to phenotype (e.g. human disease). Derivation of such interactions from a functional network model is one important way systems biology could impact applied genomics.

Current approaches to *in silico *analysis of essentiality and epistasis in metabolism employ flux balance analysis (FBA), a linear programming (LP) based approach that optimizes biomass production subject to a given combination of capacity constraints on reactions [3]. Applying a "brute force LP" approach, one can exhaustively test all combinations of single, double, triple etc. *in silico *mutants. This approach, though applicable to analysis of low-order (≤ 4) knockout combinations, is untenable for higher-order mutant combinations [1]. For example, a network of 1000 reactions would require over 10^14 ^linear programs to exhaustively test all possible quintuple-knockout combinations.

A more "elegant" approach for probing essentiality in metabolic networks is the minimal cut set (MCS) algorithm of Klamt and Gilles [4]. This algorithm employs the elementary modes (EM) of a metabolic network to uncover minimal cut sets, or minimal sets of reactions whose knockout disables a particular objective reaction. Underlying the MCS algorithm is the principle that a minimal cut set *R *for an objective reaction *j *is a minimal set of reactions that intersects all *j*-containing EM [5]. As a result, MCS can be enumerated for a network through the simple identification of "minimal hitting sets" for *j*-containing EM [6]. The main limitation of the MCS algorithm is the intractability of EM calculation for large networks (i.e. larger than 300 reactions) given current computing resources and algorithms. This limitation renders the Klamt and Gilles method inapplicable to genome-scale models.

In this paper, we demonstrate a scalable approach for uncovering high-order epistatic relationships between components of large metabolic networks. Our method is based on the analysis of *pathway fragments*, which arise as the extreme pathways of a submatrix of the stoichiometry matrix formed by taking a subset of its rows. Alternatively stated, pathway fragments are flux configurations that place only a subset of species in the system at steady state. Pathway fragments naturally emerge in the intermediate steps of the tableau algorithm for elementary mode computation [7], and are thus obtainable for a network of any size. By employing properties of the feasible flux cone, we show how knockouts, or cut sets, for an objective reaction *j *can be constructed by enumerating minimal hitting sets for *j*-containing pathway fragments. Though the resulting cut sets are not guaranteed to be minimal, they can be reduced to minimality via a second LP based step. As we show, this method offers a practical and high-yield approach for MCS computation and the study of epistasis in genome-scale metabolic networks.

We demonstrate the applicability of this approach to a genome scale metabolic model of *E. coli*, iJR904 [8]. We use our method to calculate over 11,000 MCS for biomass production. This greatly exceeds the yield of LP-based brute force and random knockout approaches in identifying such complex "essential sets". Our results represent high order epistatic relationships between components of *E. coli *metabolism and illuminate essential systems level roles of reactions in highly redundant and robust *E. coli *subnetworks.

## Results and Discussion

### Algorithm

#### Notation

ℝ is the set of real numbers, ${\mathbb{R}}_{+}^{n}$ is the set of all *n *- dimensional vectors with real and positive components, and ℝ^*m *× *n *^is the set of all *m *× *n *matrices with real entries. Given *m*, *n *∈ ℕ, we use the notation *M *= {1, ..., *m*} and *N *= {1, ..., *n*}. For a set *C*, we use |*C*| to denote its cardinality. If *A *∈ ℝ^*m *× *n *^and *U *⊆ *M*, then *A*_*U *_denotes the submatrix of *A *containing the rows with indices in the set *U*. Therefore, if *x *∈ ℝ^*n*^, *i *∈ *N*, and *U *⊂ *N*, then *x*_*i *_and *x*_*U *_∈ ℝ^|*U*| ^denote its *i*th component and the vector formed by taking components with indices in set *U*, respectively. The inequality *x *≥ 0 is interpreted componentwise, *i.e*., *x*_*i *_≥ 0, *i *= 1, ..., *n*. Each vector *x *∈ ℝ^*n *^induces a *ray*, *r *= {*αx*| *α *> 0}. We denote the set of nonzero indices of a ray *r *⊂ ℝ^*n *^as *NZ*(*r*) ⊂ *N*. The notation *r*^1 ^+ *r*^2^, where *r*^1^, *r*^2 ^are rays, refers to the usual (Minkowski) set sum.

#### Minimal cut sets

A cut set for an objective metabolic function is a set of reactions whose knockout abolishes that function [4]. Formally, a set of reactions *C *is a *cut set *for an objective reaction *j *in metabolic network *S *if and only if *v*_*j *_> 0 is feasible in the wild type (i.e. ∃*v *∈ *K*|*v*_*j *_> 0) and

(1) *v*_*C *_= 0 → *v*_*j *_= 0, ∀*v *∈ *K*.

where

(2) *K *= {*v *∈ ℝ^*n*^|*S*_*v *_= 0, *v *≥ 0}

is the *feasible flux cone *of *S*.

More generally, we call *C *⊂ *N *a cut set for an objective reaction *set J *⊂ *N *if the wild type is able to sustain flux through *every *reaction in *J *and Equation 1 is satisfied for at least one *j *∈ *J*. For example if *J *corresponds to the set of biomass component sinks in the network, a cut set for *J *will be a reaction set *C *whose knockout disables the producibility [9] of *at least *one biomass component.

A cut set *C *is *minimal *if no proper subset of *C *is a cut set. Minimal cut sets (MCS) represent "elementary failure modes" of metabolic networks [5]. MCS also capture epistatic relationships between network components that have essential roles in robust systems-level functions. In this paper, we refer to a reaction as "*k*-essential" (e.g. 1-essential, 2-essential etc.) for a metabolic objective (e.g. biomass production) if it belongs in a MCS of size *k *for that function.

Brute force LP approaches (e.g. FBA) can be applied to find MCS by exhaustively testing a collection of reaction knockouts to determine which of these "cuts" the objective reaction *j*. To test whether a reaction set *C *is a cutset for *k*, one employs an LP to determine the feasibility of *v*_*j *_> 0 subject to *v*_*C *_= 0, *v *∈ *K*. MCS can also be identified as "minimal hitting sets" of elementary modes [10]. In combinatorics, a *hitting set *of a collection of sets C, each taken from a universe of items *U*, is a set *H *⊂ *U *that intersects every set in C. In this paper, we refer to a set *H *⊂ *N *as a hitting set for a collection of *vectors E *⊂ ℝ^*n *^if *H *intersects the nonzero components of *r *for every *r *∈ *E*. *H *is a *minimal hitting set *for *E *if none of its subsets are hitting sets.

The results of Klamt and Gilles show that an MCS for an objective reaction *j *is a minimal hitting set of the collection of all elementary modes that employ *j *[4,10]. This network-based cut set criterion follows from the fact that if one knocks out a set of reactions that disables all *j*-containing elementary modes, then one will constrain the flux through reaction *j *to 0 in all feasible fluxes. These results directly extend to extreme pathways, which are equivalent to the elementary modes of an irreversible metabolic network [11]. Namely, an MCS for reaction *j *is a minimal hitting set of all the *j*-containing extreme pathways. We choose the extreme pathway convention in this paper because it tends to be a more compact representation of the pathway structure of metabolism [12].

Both the brute-force LP approach and the MCS algorithm of Klamt and Gilles have deep limitations in discovering genome-scale MCS. The size of most genome-scale metabolic networks (> 500 – 1000 reactions) renders brute-force LP approaches practical only for finding low cardinality MCS (*k *≤ 2), though high performance computing and restriction of the search to a subset of model reactions can extend this limit to *k *≤ 4 [1]. The MCS algorithm of Klamt and Gilles is not applicable to the genome-scale due to its dependence on elementary mode computation, which is only feasible for small metabolic networks (i.e. less than 300 reactions) [4,10,11,13].

In this paper we introduce *NetKO*, a three-step procedure for generating genome scale MCS. In the first step, we use the tableau algorithm to generate a collection of *pathway fragments *for the metabolic network. In the second step, we compute a collection of cut sets for each reaction *j *∈ *J *through the analysis of pathway fragments. In the third step, we use pairwise set comparisons and LP to generate a collection of MCS for the objective reaction set *J*.

#### Extreme pathways

The feasible flux cone *K *of a metabolic network *S *is the set sum of a finite and unique collection of extreme rays *E*(*K*), called *extreme pathways *[14]. The standard tableau algorithm for computing extreme pathways *E*(*K*) associated with stoichiometry matrix *S *is an iterative procedure that computes the extreme rays *E*(*K*^*i*^) for a series of polyhedral cones *K*^*i *^⊂ ℝ^*n*^, *i *∈ {0, ..., *m*} given by:

(3) $$K^{i}=\{v|S_{M_{i}}v=0,v\geq 0,M_{i}=\{1\ldots i\}\}.$$

The algorithm is seeded with the initial cone *K*^0 ^= ${\mathbb{R}}_{+}^{n}$. The initial collection of generators *E*(*K*^0^) consists of rays induced by the Euclidean basis vectors *e*^*j *^∈ ℝ^*n*^. At each iteration *i *∈ {1, ..., *m*}, the generators *E*(*K*^*i*^) are computed from the analysis of rays in *E*(*K*^*i *- 1^) in three steps.

In the first step, each ray in *E*^*i*-1 ^is tested to determine whether it belongs to the hyperplane *S*_*i*_*v *= 0. Rays in *E*(*K*^*i *- 1^) that belong to this hyperplane are added to the collection *E*(*K*^*i*^). In the second step of iteration *i*, the algorithm considers each ray pair in *E*(*K*^*i *- 1^) that lies on opposite sides of the hyperplane *S*_*i*_*v *= 0. The intersection of the set sum of each ray pair with *S*_*i*_*v *= 0 induces a new ray that lies within the cone *K*^*i*^. Each such ray is added to the collection *E*(*K*^*i*^).

The third and final step of each iteration *i *involves removal of non-extreme rays from *E*(*K*^*i*^). This is implemented by comparing the sparsity patterns of each ray pair in *E*(*K*^*i*^) and removing any ray *r *for which there exists an *r*^' ^such that *NZ*(*r*^'^) ⊂ *NZ*(*r*). Following iteration *m*, the tableau algorithm terminates, having computed the extreme rays of polyhedral cone *K *= *K*^*m*^. However, given the computational complexity of the third step in each iteration, the tableau algorithm fails to terminate (i.e. reach iteration *m*) for most genome-scale metabolic networks.

#### Pathway fragments enable a "relaxed" cut set criterion

Each iteration *i *of the tableau algorithm produces a collection of rays *E*(*K*^*i*^) ⊂ ℝ^*n*^, which we refer to as *pathway fragments*. Each pathway fragment in *E*(*K*^*i*^) obeys the quasi steady state assumption for the subset *M*_*i *_⊆ *M *of species in the system $\dot{x}$ = *Sv*, *v *≥ 0. Though a collection of pathway fragments contains incomplete information about metabolic network dynamics, we will show that it offers much insight into its failure modes. We exploit this property in our network-based genome-scale knockout design approach. Each pathway fragment collection *E*(*K*^*i*^) contains the extreme rays of the cone *K*^*i *^in Equation 3.

Applying Klamt and Gilles' criterion to Equation 3, a minimal hitting set *C *for the *j*-containing rays in *E*(*K*^*i*^) is an MCS for reaction *j *in the "relaxed" system $S_{M_{i}}v$ = 0, *v *≥ 0. This means that *C *is a minimal. set of reactions satisfying the relation:

(4) *v*_*C *_= 0 ⇒ *v*_*j *_= 0, ∀*v *∈ *K*^*i*^

**Figure 1:**
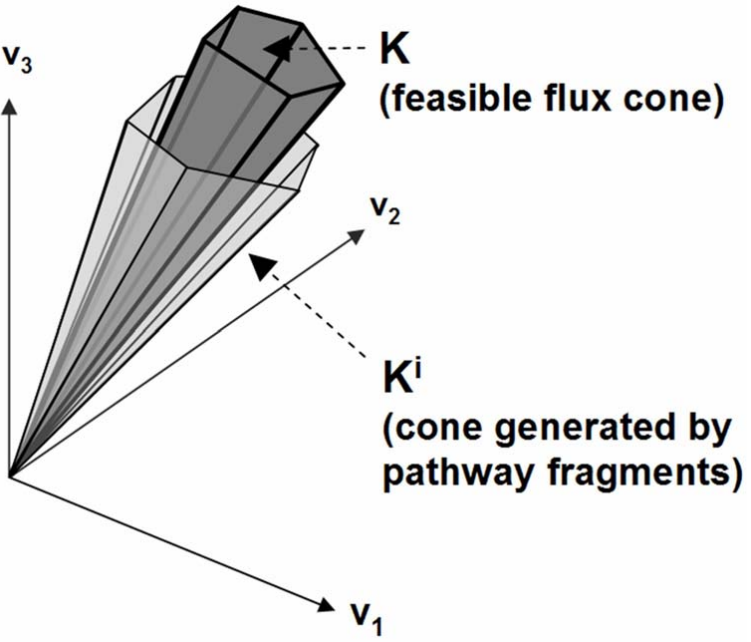
**Pathway fragments enable a "relaxed" cut set criterion**. The feasible ux cone *K *of a metabolic network is contained inside the cone *K*^*i *^generated by the pathway fragment collection *E*(*K*^*i*^) obtained from any iteration *i *of the tableau algorithm. Because of this property, the knockout of a reaction set *R *that intersects all *j*-containing pathway fragments in *E*(*K*^*i*^) will be guaranteed to "cut" the ux through an objective reaction *j*.

Comparison of Equations 2 and 3 shows that the cone *K*^*i*^generated by a pathway fragment collection *E*(*K*^*i*^) is an *overapproximation *of the final feasible ux cone *K *(Figure 1). Formally,

(5) *K *⊆ *K*^*i*^.

As a result, any flux configuration that is infeasible in *K*^*i*^will also be infeasible in *K*. Alternatively stated, if *C *satisfies Equation 4 then it also satisfies Equation 1. Thus, *C *is a cut set for *j *in the full system *Sv *= 0, *v *≥ 0.

In this section, we have proven a "relaxed" version of Klamt and Gilles' cut set criterion. Namely, we have shown that a minimal hitting set for a collection of *j*-containing pathway fragments is a cut set for *j *in a metabolic network *S*. As a result, the problem of computing cut sets for an objective reaction *j *can be reduced to the enumeration of minimal hitting sets for the *j*-containing pathway fragments. The latter can be achieved by iterating through the *j*-containing pathway fragments via the procedure *MinHit *(Table 3), described in the implementation section. As we will show in the results, application of this "relaxed" pathway-fragment based cut set criteria enables network-based knockout design on the genome-scale.

#### Post-processing for non-minimal cut sets

Our "relaxed" cut set criterion is able to generate genuine cut sets for the *entire *metabolic network *S *despite employing only a traversal across the *subset M*_*i *_of metabolites. However, the incompleteness of this traversal makes our criterion *sufficient but not necessary *for determining whether a reaction set *C *constitutes a cut set for reaction *j*. This results in two caveats regarding the "quality" of cut sets obtained from the analysis of pathway fragments: 1) not all cut sets are guaranteed to be found and 2) cut sets that are found are not guaranteed to be minimal. To fully reduce cut sets generated by *MinHit *to minimality, we apply an LP based post-processing step *Reduce *(Table 4) to 1) checks minimality of cut sets and 2) (if necessary) reduces them to their minimal subsets. We describe *Reduce *in more detail in the Methods section.

There may be additional sources of non-minimality when one pursues MCS for a *set *of objectives *J *(e.g. the set of biomass sinks). In this case a cut set obtained for one reaction *j*^1 ^∈ *J *may be a subset of a cut set obtained for a second reaction *j*^2 ^∈ *J*. Both may be MCS for their respective objectives, however one (or both) may be non-minimal with respect to the objective set *J*. For example, if *J *corresponds to the set of biomass sinks in the network, an MCS for L-isoleucine production may be built by adding reactions to an L-threonine MCS. Such non-MCS may be immediately pruned through a pair-wise comparison of cut sets generated by *MinHit *for the various individual sinks in *J*, and removal of sets for which there exists a subset.

#### Guiding the tableau algorithm to maximize pathway fragment quality

As may be intuitively expected, the quality of cut sets obtained with our approach directly depends on the "quality" of pathway fragments. At each iteration, the tableau algorithm "learns" more about network constraints imposed by the steady state requirement for each individual species. As a result, later iterations of the tableau algorithm yield more informative pathway fragments. In other words, pathway fragments gathered from an early iteration of the algorithm are "naive" and will yield fewer cut sets that are farther from being minimal. Conversely, "mature" pathway fragments gathered from a later iteration will yield larger numbers of cut sets that are closer to being minimal.

A simple approach that we apply to maximize the iteration *i *reached by the tableau algorithm is the "local greedy" optimization strategy described by Bell and Palsson [15]. This strategy is applied after each iteration of the tableau algorithm to choose the "cheapest" metabolite for the following iteration. The cost of each metabolite is computed as the number of pathway fragments at that iteration that consume that metabolite multiplied by the number of pathway fragments that produce that metabolite. This metabolite ordering strategy can be conceptually understood as a network traversal that begins with the least connected metabolites (e.g. network dead ends) and saves highly connected metabolites (e.g. ATP, water) for later iterations. Though we exclusively use this strategy for this study, we will mention several alternative metabolite ordering approaches that may be used for network-based genome-scale knockout design later in the discussion.

### Network-based approach yields 11,706 complex essential sets in *E. coli*

To test our approach, we examined failure modes for rich media biomass production in the *E. coli *IJR904 genome-scale metabolic network model. In rich media, all extracellular nutrients are made available, resulting in many redundant pathways for biomass biosynthesis. This redundancy makes biomass production very difficult to suppress via single or double knockouts.

**Figure 2:**
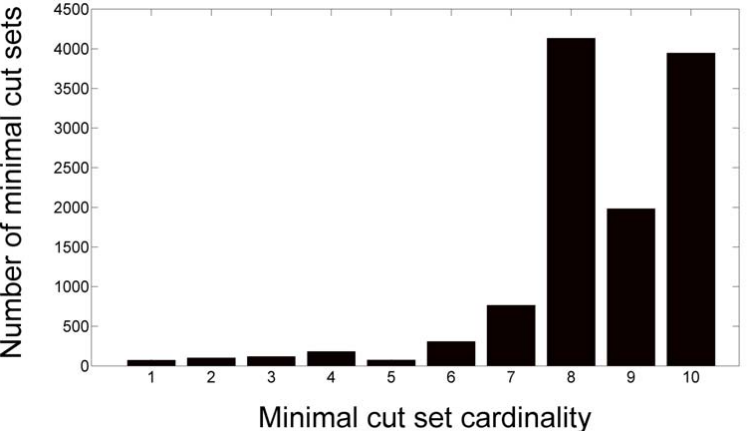
**NetKO uncovers high-cardinality minimal cut sets**. Our network-based genome-scale knockout design approach preferentially discovers high cardinality minimal cut sets for biomass production: 11,218 of the 11,706 minimal cut sets discovered are of cardinality 5 or above. These minimal cut sets represent high-order epistasis between parallelized metabolic network components.

Our network based analysis approach found 11,706 MCS for biomass production in rich media. These MCS target 36 of 49 biomass components and employ 355 of the 1324 possible non-sink reactions in the *E. coli *network. As shown in Figure 2, most of these 11,706 essential sets have high cardinality (> 5 reactions). The vast majority (11343) of the MCS carry out a precise "surgical strike" on biomass production, targeting the synthesis of only a single component. Biomass components most often targeted by these MCS are L-threonine (10655 MCS), dTTP (297 MCS), 5-methyl-THF (216 MCS), FAD (197 MCS), and dGTP (190 MCS). All 11,706 MCS and their biomass targets are included as Supplementary Data [see Additional File 1.

#### Essential sets reveal epistasis between diverse *E. coli *subsystems

**Figure 3:**
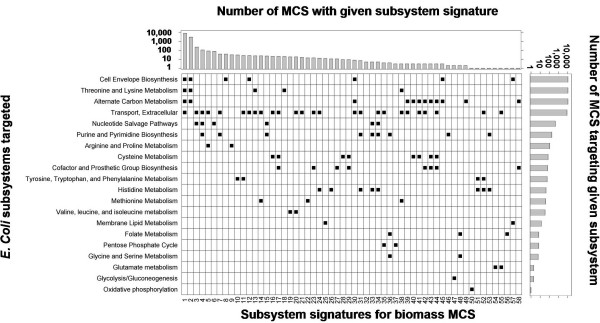
**MCS target multiple *E. coli *subsystems**. Minimal cut sets (MCS) for biomass production target 23 of 30 *E. coli *reaction subsystems. The most often targeted subsystems are "Cell Envelope Biosynthesis", "Threonine and Lysine Metabolism", "Alternate Carbon Metabolism", and "Extracellular Transport". 7 subsystems are not targeted by any MCS, including 'Alanine and aspartate metabolism', 'Anaplerotic reactions', 'Pyruvate metabolism', and 'Citrate Cycle (TCA)'. Subsystem annotations are taken from the original *E. coli *iJR904 model annotation [8]. For compactness, subsystems "Unassigned", "Putative", and "Putative Transporters" were not included in the subsystem signatures shown in the above figure.

The original model annotation of *E. coli *iJR904 groups reactions into 30 "subsystems" [8]. The MCS obtained from network analysis span 23 of these 30 reaction subsystems in the *E. coli *metabolic network. Superimposition of the MCS on the subsystem reaction classification yields 58 unique "subsystem signatures", which are shown in Figure 3. Analyzing these signatures shows that only 289 of the MCS discovered with our approach target a single subsystem, while the vast majority (10838) target three or four subsystems. The most prevalent (7720 MCS) of these multivalent subsystem signatures target the combination of "Cell Envelope Biosynthesis", "Threonine and Lysine Metabolism", "Alternate Carbon Metabolism", and "Extracellular transport". MCS with such signatures clearly represent complex interactions between functionally disparate and parallel portions of the *E. coli *metabolic network.

#### NetKO efficiently uncovers complex interactions in *E. coli *metabolic network

To demonstrate the utility of NetKO, we benchmarked it against two standard *in silico *metabolic knockout analysis approaches: brute-force LP and random knockout. For brute-force LP, we tested all single and double knockouts. In our random knockout approach, we sampled 250,000 random reaction sets of cardinality 10. For each such set, we determined whether its knockout cut the production of biomass and reduced it to minimality with respect to biomass production (via *Reduce*).

Comparing the results (Table 1) we found that *NetKO *was able to yield a larger number of MCS that targeted more reactions in the network than either brute force or random knockout approaches. Table 1 shows that our network-based genome-scale knockout design yielded over 50 times as many MCS as both brute-force and random KO approaches. Furthermore, MCS obtained using our network-based approach implicate a larger number of reactions as *k *< 10-essential for biomass production for rich media *E. coli*. Though we employ different cardinality limits for the network based (*k *≤ 10) and brute force (*k *≤ 2) approaches in this benchmark, we note that these results reflect a similar order of magnitude of LP operations (115,243 vs 487,671), resulting in higher return for a similar expenditure of computational resources. In comparison to the random approach, which also tests reaction sets of cardinality 10, we note that *NetKO *employs two orders of magnitude fewer linear programs than a random knockout approach (113,989 vs 7,221,149) while yielding many more high-order interactions.

**Table 1:** Benchmarking Results

**Property**	**Brute-force LP (k = 2)**	**Random KO + Reduce (k = 10)**	**NetKO (k = 10)**
Number of MCS found	215	223	11,706
Highest Cardinality MCS Obtained	2	3	10
Reactions included in at least one MCS	263	235	355
Number of biomass components targeted	38	41	36
Number of linear programming steps	487,670	7,221,149	113,989

Comparison of our network-based genome-scale knockout design approach (NetKO) with two LP-based methods: The standard genome-scale method for MCS computation for all single and double knockouts (Brute Force LP) and a random knockout approach combined with reduction to minimality for 250,000 cardinality 10 reaction sets (Random KO + reduce).

Comparison of brute KO and *NetKO *results show that in many ways the two approaches are complementary (Table 1). For example, *NetKO *is not guaranteed to find *all *cut sets of a given cardinality: it misses 99 of the 215 MCS found with brute-force LP. Furthermore, MCS obtained using brute-force LP cover an alternate set of reactions and biomass components than MCS discovered using *NetKO*. This occurs because *NetKO *is biased towards finding high-cardinality MCS. Though the number of reactions belonging to Brute-force LP and *NetKO *derived MCS are the same, we note that *NetKO *establishes *k *< 10-essential roles for 184 *E. coli *reactions that have no 1- or 2- essential role in biomass production. The differences in biomass targets also reflect the fact that brute force LP is biased towards biomass components targeted by low-cardinality cut sets (e.g. LPS). Because of these differences, one can use "brute force LP" results to determine general network properties regarding the rate of interaction between genes for a given degree of epistasis *k *[2], which cannot be gleaned from the results of *NetKO*. Conversely, unlike brute force LP, one can use *NetKO *to uncover very high-order epistatic relationships for robust network functions.

Our random KO results demonstrate quite strikingly how difficult it is to find high order MCS in the *E. coli *metabolic network (Table 1). Though many (52%) of the 250,000 random reaction sets of size 10 cut biomass production, all of these cut sets are built from a relatively small number (223) of size 1 to 3 MCS. The rarity of high cardinality MCS in these results is a testament to the efficacy of *NetKO *in exploiting network structure for knockout design. Additionally, the difference between the size of these cut sets and the size of MCS they contain shows why it is so expensive (requiring over 7 million LP's) to reduce these random high-cardinality cut sets to minimality. In contrast, cut sets generated through pathway fragment analysis in *NetKO *are much closer to minimality, requiring much fewer LP's in the *Reduce *computational step.

#### Minimal cut sets targeting L-threonine reveal a robust 41 reaction biosynthetic subnetwork

In *E. coli *iJR904, L-threonine production is invulnerable to all single or double knockouts. However, *NetKO *discovers 10655 MCS that target the production of this amino acid: all but 10 of these L-threonine targeting MCS employ more than 5 reactions, and 9725 involve 8 or more reactions.

**Figure 4:**
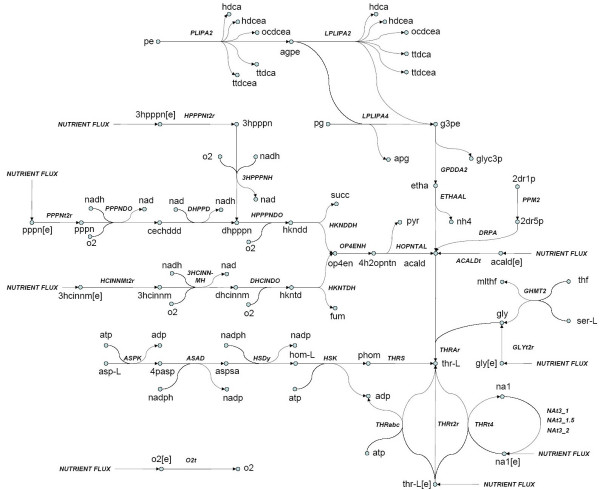
**MCS uncover robust L-threonine biosynthesis subnetwork**. 10,655 minimal cut sets discovered using our approach target L-threonine synthesis via this 41 reaction subnetwork of *E. coli *metabolism. None of these reactions are 1- or 2-essential for L-threonine production, though 7 participate in size 1 or 2 MCS for peptidoglycan and L-methionine production. Though many of these cut sets are complex (i.e. involve 7 or more reactions) they can be intuitively understood as simultaneous attacks on a few distinct pathways that converge on L-threonine (thr-L) synthesis. For ease of presentation, we omit water and proton in the reaction depictions. Please refer to Table 2 for explanation of metabolite and reaction abbreviations. Please refer to the text for further discussion.

Interestingly, these 10655 genome-scale MCS arise exclusively from a 41 reaction subnetwork of *E. coli*, which is depicted in Figure 4. Furthermore, only 7 of the reactions in this subnetwork (ASAD, ASPK, DAPabc, HSDy, METabc, 26dap-M and met-L nutrient fluxes) have 1- or 2-essential roles in rich media biomass production; however, low cardinality MCS containing these reactions target peptidoglycan and L-methionine rather than L-threonine production. Our MCS results thus rigorously establish novel systems-level roles for all 41 of these reactions. These results link the systems-level process of L-threonine production in *E. coli *to the functioning of a highly parallelized biosynthetic subnetwork. Epistasis between components of this subnetwork only become apparent through the analysis of high-order knockouts. The L-threonine subnetwork shown in Figure 4 is composed of reactions from the 'Alternate Carbon Metabolism', 'Cell Envelope Biosynthesis', 'Putative Transporters', 'Threonine and Lysine Metabolism', and 'Transport, Extracellular' subsystems. Though it is responsible for a large number of MCS, this subnetwork can be easily understood as the convergence of several pathways linking extracellular carbon sources, cell membrane components, amino acids, and other intracellular species to L-threonine. As we show below, analysis of these pathways yields biochemically intuitive rationale for why these reaction subsets emerge as biomass MCS.

**Table 2:** List of Abbreviations

Abbreviation	Full Name	Abbreviation	Full Name
2dr1p	2-Deoxy-D-ribose 1-phosphate	agpe_EC	acyl-glycerophospoethanolamine (E. coli) **
2dr5p	2-Deoxy-D-ribose 5-phosphate	apg_EC	acyl phosphatidylglycerol (E. coli) **
3HCINNMH	3-hydroxycinnamate hydroxylase	asp-L	L-Aspartate
3HPPPNH	3-(3-hydroxy-phenyl)propionate hydroxylase	aspsa	L-Aspartate 4-semialdehyde
3hcinnm	3-hydroxycinnamic acid	atp	ATP
3hcinnm [e]	3-hydroxycinnamic acid (Extracellular)	cechddd	cis-3-(3-carboxyethyl)-3,5-cyclohexadiene-1,2-diol
3hpppn	3-(3-hydroxy-phenyl)propionate	dhcinnm	2,3-dihydroxicinnamic acid
3hpppn [e]	3-(3-hydroxy-phenyl)propionate (Extracellular)	dhpppn	3-(2,3-Dihydroxyphenyl)propanoate
4h2opntn	4-Hydroxy-2-oxopentanoate	etha	Ethanolamine
4pasp	4-Phospho-L-aspartate	fum	Fumarate
ACALDt	acetaldehyde reversible transport	g3p	Glyceraldehyde 3-phosphate
ASAD	aspartate-semialdehyde dehydrogenase	g3pe	sn-Glycero-3-phosphoethanolamine
ASPK	aspartate kinase	gly	Glycine
DHCINDO	2,3-dihydroxycinnamate 1,2-dioxygenase	glyc3p	Glycerol 3-phosphate
DHPPD	2,3-dihydroxyphenylpropionate dehydrogenase	h	H+
DRPA	deoxyribose-phosphate aldolase	h2o	H2O
ETHAAL	Ethanolamine ammonia-lyase	h [e]	H+ (Extracellular)
GPDDA2	Glycerophosphodiester phosphodiesterase	hdca	Hexadecanoate (n-C16:0)
HCINNMt2r	3-hydroxycinnamic acid transport	hdcea	hexadecenoate (n-C16:1)
HKNDDH	2-hydroxy-6-ketonona-2,4-dienedioic acid hydrolase	hkndd	2-Hydroxy-6-oxonona-2,4-diene-1,9-dioate
HKNTDH	2-hydroxy-6-ketononotrienedioate hydrolase	hkntd	2-hydroxy-6-ketononatrienedioate
HOPNTAL	4-hydroxy-2-oxopentanoate aldolase	hom-L	L-Homoserine
HPPPNDO	2,3-dihydroxypheylpropionate 1,2-dioxygenase	na1	Sodium
HPPPNt2r	3-(3-hydroxyphenyl)propionate transport	na1 [e]	Sodium (Extracellular)
HSDy	homoserine dehydrogenase (NADPH)	nad	Nicotinamide adenine dinucleotide
HSK	homoserine kinase	nadh	Nicotinamide adenine dinucleotide – reduced
LPLIPA2	Lysophospholipase L (acyl-glycerophosphoethanolamine)	nadp	Nicotinamide adenine dinucleotide phosphate
LPLIPA4	Lysophospholipase L (acyl transfer to phosphatidylglycerol)	nadph	Nicotinamide adenine dinucleotide phosphate – reduced
NAt3_1	sodium proton antiporter (H:NA is 1:1)	nh4	ammonium
NAt3_1.5	sodium proton antiporter (H:NA is 1.5)	o2	O2
NAt3_2	sodium proton antiporter (H:NA is 2)	o2 [e]	O2 (Extracellular)
O2t	o2 transport (diffusion)	ocdcea	octadecenoate (n-C18:1)
OP4ENH	2-oxopent-4-enoate hydratase	op4en	2-Oxopent-4-enoate
PLIPA2	Phosphlipase A (phosphatidylethanolamine)	pe_EC	Phosphatidylethanolamine (Ecoli) **
PPM2	phosphopentomutase 2 (deoxyribose)	pg_EC	Phospatidylglycerol (Ecoli) **
PPPNDO	Phenylpropanoate Dioxygenase	phom	O-Phospho-L-homoserine
PPPNt2r	3-phenylpropionate transport	pi	Phosphate
THRAr	Threonine Aldolase	pppn	Phenylpropanoate
THRS	threonine synthase	pppn [e]	Phenylpropanoate (Extracellular)
THRabc	L-threonine transport via ABC system	pyr	Pyruvate
THRt2r	L-threonine reversible transport via proton symport	succ	Succinate
THRt4	L-threonine via sodium symport	thr-L	L-Threonine
acald	Acetaldehyde	thr-L [e]	L-Threonine (Extracellular)
acald [e]	Acetaldehyde (Extracellular)	ttdca	tetradecanoate (n-C14:0)
adp	ADP	ttdcea	tetradecenoate (n-C14:1)

List of metabolites, reactions, and their abbreviations.

The simplest of the 10655 L-threonine targeting MCS discovered using our approach consist of reactions immediately upstream of L-threonine in this subnetwork. As shown in Figure 4, the most direct sources of L-threonine in the intracellular environment are three transport reactions that couple the influx of L-threonine to the import of sodium (THRt4), the import of proton (THRt2r), and the hydrolysis of ATP (THRabc). L-threonine is also synthesized from L-aspartate via a pathway that employs L-homoserine as an intermediate. The third major source of L-threonine is via the threonine aldolase reaction (THRAr), whose substrates are acetaldehyde and glycine.

The most intuitive of these MCS consists of the three L-threonine transporters (THRt4, THRt2r, THRabc) in combination with threonine synthetase (THRS) and threonine aldolase (THRAr). Variants of this MCS replace THRS with aspartate-semialdehyde dehydrogenase (ASAD), homoserine kinase (HSK), homoserine kinase (HSK), or aspartate kinase (ASPK). A second set of variants replaces the three L-threonine transporters with the L-threonine nutrient flux. 5 MCS apply an interesting variation upon this theme by replacing the sodium dependent THRabc transporter with either the sodium nutrient flux or inhibition of the three sodium proton antiporters (NAt3_2, NAt3_1.5, NAt3_1). Because of their minimality, the knockout of *any *subset of these MCS will allow L-threonine production to be maintained in this *silico *strain. For example, knockout of L-threonine transport and threonine-aldolase will allow L-threonine to be produced from L-aspartate via the pathway shown in the bottom left of Figure 4. Alternatively stated, the minimality of these cut sets attests to the fact that the wild type *in silico *can employ *any one *of these parallel pathways for L-threonine biosynthesis.

A large number of MCS discovered using our approach target L-threonine biosynthesis by disabling pathways upstream of acetaldehyde. As shown in Figure 4, acetaldehyde is directly brought into the cell from the nutrient media via a transport reaction. Acetaldehyde is also supplied by the breakdown of phosphatidylethanolamine and phosphatidylglycerol, which are cell membrane components. Acetaldehyde is also formed from extracellular phenylpropanoate, 3-(3-hydroxy-phenyl)propionate, and 3-hydroxycinnamic acid, which are substrates for the synthesis of intracellular 2-Oxopent-4-enoate. The latter is transformed into acetaldehyde via 2-oxopent-4-enoate hydratase (OP4ENH) and 4-hydroxy-2-oxopentanoate aldolase (HOPNTAL), producing pyruvate as a byproduct. Another source of acetaldehyde is via the breakdown of deoxyribose sugars via the reactions deoxyribose phosphate aldolase (DRPA) and phosphopentomutase 2 (PPM2). The more complex L-threonine targeting MCS replace the THRAr reaction in the simple MCS described above with a combination of the reactions responsible for acetaldehyde biosynthesis. One such combination is DRPA, HOPNTAL, ACALDt, and ethanolamine ammonia-lyase (ETHAAL). A variation of this MCS interchanges ETHAAL in the latter combination with Phospholipase A2 (PLIPA2). Another combination replaces HOPNTAL with the combination of phenylpropanoate, 3-(3-hydroxy-phenyl)propionate, and 3-hydroxycinnamic acid transporters. 160 MCS target L-threonine production through a unique mechanism that involves the repression of the oxygen nutrient flux. Like the MCS discussed in the previous paragraph, these sets include reactions that involve acetaldehyde formation from cell membrane phospholipids, ribose sugar breakdown, and acetaldehyde transport. However unlike the previously described sets, these MCS exploit the oxygen-dependence of the pathways facilitating acetaldehyde production from extracellular phenylpropanoate, 3-(3-hydroxy-phenyl)propionate, and 3-hydroxycinnamic acid. As shown in Figure 4, the reactions 3-(3-hydroxy-phenyl)propionate hydroxylase (3HPPPNH), Phenylpropanoate Dioxygenase (PPPNDO), 3-hydroxycinnamate hydroxylase (3HCINNMH), 2,3-dihydroxycinnamate 1,2-dioxygenase (DHCINDO), and 2,3-dihydroxyphenylpropionate 1,2-dioxygenase (HPPPNDO) all depend on the presence of oxygen as a substrate.

The L-threonine subnetwork described in this section demonstrates a biologically intuitive pathway-like structure. The ease with this network can be understood testifies to the ability of *NetKO *to unravel the link between network components and systems-level functions. We further note that unlike manual pathway annotations, this subnetwork is derived purely through the analysis of flux dynamics in the genome-scale metabolic model. As a result, it serves as a rigorous and objective distillation of *E. coli *iJR904's behavior with respect to a particular function, i.e. L-threonine production.

### Applications

#### Drug design

MCS for essential metabolic objectives (e.g. biomass) represent potential targets for drug development. Though the model employed in this study represents a non-pathogenic strain of *E. coli*, there are numerous genome-scale metabolic reconstructions of pathogenic microbes in which such an approach could be used to reveal drug targets. Such organisms include *S. aureus*, *H. pylori*, and *B. anthracis *[16-19]. Furthermore, high-order MCS could be applied to models of human metabolic networks to define novel anti-proliferative chemotherapeutic regimens [20].

#### Epistatic candidate gene models for genetic association

MCS correspond to subsets of genes whose perturbation or knockout is predicted to effect a network-level phenotype. Such a gene subset provides a reasonable "epistatic" candidate hypothesis for a genetic association study that explore links between genotype and phenotype, e.g. for human disease. Current approaches to whole genome association studies employ a unsupervised approach that test all possible loci for association with a phenotype. Given the high dimensionality of genotypic (e.g. SNP) data, these studies are limited by computational resources and statistical power to searching for binary gene-gene interactions. A "systems-based candidate-gene" approach based on *NetKO *could provide a powerful approach to apply "a posteriori" biological knowledge to study the role of epistasis in disease. Namely, one could employ *NetKO *to generate hypothesis in the form of MCS on a given pathway model and test such hypotheses against genotype-phenotype data.

#### Elucidating systems-level roles of reactions in metabolism

MCS discovered using our approach cluster into subnetworks that link individual reactions to their systems-level roles. In contrast to the daunting complexity of the entire metabolic network, these subnetworks have an intuitive pathway structure that can be understood by simple inspection. The close dual relationship between the failure and function of of metabolism has been previously outlined by Klamt [5]. This dual relationship suggests that these local pathways are likely "projections" of genome-scale extreme pathways/elementary modes onto these important reaction subsets. This hypothesis could be verified by applying the method of Urbanczik and Wagner to compute generators for the projection of the flux cone *K *onto these reaction subsets [21].

Many of our MCS reveal essential roles for reactions whose function is normally obscured by metabolic network redundancy. Deutscher *et al*. refer to this generalized concept of essentiality as "*k*-robustness" in their investigations of multiple knockouts in yeast metabolism [1]. Each complex MCS of cardinality *k *identified by our approach is conceptually equivalent to Deutscher *et al*.'s "essential set". High cardinality MCS represents an instance of a higher-order genetic or biochemical interaction between several reactions, enzymes, and genes that is mediated by the metabolic network. Similar to the results of Deutscher *et al*., we find that application of this generalized notion of essentiality significantly increases the number of *E. coli *reactions that can be thought of as participating in "essential processes" (as defined by biomass production).

The use of a genome-scale model to find essential links between genes and systems-level processes exemplifies a novel "network-based" approach to functional gene annotation. Shlomi *et al*. show that such an approach may be better equipped to capture functional features such as condition-dependence when compared to traditional structured vocabulary-based annotation schemes like Gene Ontology [22]. *NetKO *provides a powerful tool for this kind of annotation by helping uncover functional links that are normally buried by redundant pathways.

#### Iterative model building and refinement

Our approach can be applied in an iterative model building protocol that combines *in vivo *experiment and *in silico *analysis.

One specific application of *NetKO *is the simple validation of model completeness and accuracy on the genome-scale. A more profound application is to unravel the systems-level roles of individual reactions, in particular those participating in robust and redundant network functions.

Fundamentally, each MCS can be understood to suggest several important *in vivo *model validation experiments. The first corresponds to the knockout of that MCS and the subsequent lethality prediction. If the *in vivo *knockout fails to be lethal, then this suggests that either there is additional redundancy in the pathway structure of the network or the specific biomass target of that MCS is non-essential for growth [9]. Once an MCS is verified as being *in vivo *lethal, an interesting set of experiments can be performed that test the contributions of individual reactions to the function disabled by a given MCS. To do so, one would design knockouts that disable *all but one *of the reactions in that MCS. Such a perturbation would "pinch" the network, allowing only extreme pathways that utilize the remaining reaction in that MCS. As a result, the flux through that reaction would be predicted to become a simple linear multiple of the growth rate (determined only by the steady state intracellular concentration of the target biomass component and its fractional contribution to biomass).

For example, the cysteine transporter (CYSabc), taurine transporter (TAURabc), biotin synthase (BTS2), and the sulfate transporter (SULabc) form an MCS for biomass by targeting L-cysteine production. Knockout of CYSabc, TAURabc, and BTS2 would be predicted by this network model to force all fluxes destined for L-cysteine production through the sulfate transporter (SULabc). According to the model, the rate of uptake of sulfate in this triple knockout will be in direct proportion to the flux through the L-cysteine sink, which corresponds to the growth-mediated dilution of L-cysteine and its consumption by protein synthesis. Such a discrete "pinching" experiment would determine whether the sulfate transporter is sufficient to sustain L-cysteine production. Extension of such an experimental paradigm to analyze further subsets of complex MCS at intermediate levels of inhibition could be used to quantitatively identify the "marginal" contributions of individual reactions/pathways to systems-level functions.

### Improving MCS yield

*NetKO *discovers valid genome-scale MCS using only partial information regarding metabolic network dynamics. However, because it only employs partial information, it is not guaranteed to find *all *MCS for a given objective. For example, despite detecting many high-order interactions, our approach fails to detect nearly half of the cardinality *k *≤ 2 MCS. Furthermore, the biomass targeting MCS that we find utilize only 355 of the potential 1324 reactions in the model. Though this may suggest that the remaining reactions in the network participate only in highly redundant (i.e. *k *> 10-robust) network functions, it is also likely that our approach fails to find a significant number of *k *≤ 10 MCS.

As mentioned earlier in the paper, the yield of our method depends directly on the "quality" of pathway fragments generated from the application of the tableau algorithm to the stoichiometric matrix. Specifically, the number of reactions belonging to a biomass-targeting MCS identified by our approach corresponds to the number of reactions that share a pathway fragment with a biomass sink. Improving the reaction "span" of these biomass-containing pathway fragments would increase the number of reactions employed by biomass-targeting MCS as well as the total yield of MCS.

One approach for improving the yield of MCS would be to achieve additional incremental improvement in the progress of the tableau algorithm. Each iteration of the tableau algorithm processes the steady state requirements of another metabolite in the network, and thus "learns" more about the constraints imposed by network structure. By applying more raw computational power in the form of memory and processor speed, one could achieve several more iterations that would link more reactions to the collection of biomass-containing pathway fragments. In addition, one could apply high performance computing employing a parallel out-of-core approach to achieve additional progress [23]. Though such approaches have been investigated in the past with the goal of completing the entire extreme pathway computation for a genome-scale metabolic network, our approach offers an incentive for performance-improving modifications that achieve even incremental progress in the tableau algorithm.

A more finessed approach for improving MCS yield may be to use an alternative metabolite "traversal" strategy for the tableau algorithm. In our implementation, we use a "local greedy optimization" strategy that chooses the "cheapest" next metabolite using a generic evaluation of each metabolite's "cost" [15]. Though this approach may be optimal for achieving total progress in the algorithm (i.e. with respect to iterations), it may not be the best approach of generating MCS for a particular metabolic objective. A simple approach to maximize the number of cut sets is to ensure that the pathway fragment algorithm traverses the substrates of the objective reaction of interest. In the case of the biomass reaction, it may not be computationally feasible to generate a pathway fragment collection that traverses all substrates simultaneously, but one may compute cut sets for individual biomass components through separate pathway fragment runs. More generally one would like to guide the tableau algorithm from the out set to choose metabolites that expand the number of reactions that share a pathway fragment with the objective reaction (e.g. a biomass component). One way of achieving this is to look for "bridging" species at each iteration that link a reaction that shares pathway fragments with the objective reaction and a second reaction that does not. We are currently investigating such approaches.

### Alternative approaches

Our approach is not the first attempt at "rational" genome-scale knockout design. Maranas and colleagues have previously applied mixed integer linear programming (MILP) to design mutants that improve byproduct yield or identify "minimal reaction sets" that are capable of sustaining a given biomass optimum [24,25]. These applications of MILP approaches differ fundamentally from the knockout design problem approached in this paper. Unlike the computation of minimal reaction sets, which corresponds to finding a *maximal *number of reaction deletions that allow biomass production to be *sustained*, the computation of MCS corresponds to finding a *minimal *number of reaction deletions that cause biomass production to be *abolished*.

Though a precedent does not exist, MCS discovery could potentially be formulated as a MILP. Such a MILP would employ as its objective the minimization of the number of deleted reactions that abolish biomass production. Once an MCS is found as the solution of the MILP, additional MCS could be discovered through the execution of successive MILPs that incorporate additional constraints to prevent the rediscovery of previous optima. Such an approach is promising, however requires further investigation to address at least two major concerns: Firstly, MILPs are NP-complete, and thus scale poorly with the dimension of the search space, i.e. the number of reactions. Secondly, repeated addition of constraints to discover alternative optima may increase the "stiffness" of successive MILPs as further MCS are sought. As a result, it is *a priori *unclear that a MILP-based MCS algorithm would be able to generate as many MCS as our network based approach.

## Conclusion

We have introduced and applied a network-based method for genome-scale knockout design. Our approach identifies high-order essential epistatic interactions that are normally obscured by metabolic network redundancy. These interactions cluster along interesting and physiologically important subnetworks that allow systems-level roles of individual reactions to be precisely and intuitively captured. Our method provides a plethora of predictions that can readily translate into informative *in vivo *knockout experiments in *E. coli*. These experiments could be used for iterative model building and functional annotation of genes. Future applications of our network based knockout design approach includes analysis of the metabolic networks of pathogenic organisms and generation of epistatic candidate models for genome-wide association studies.

## Methods

### Tableau algorithm

We implement the tableau algorithm according to previously described implementations [7,15], adding a "pathway fragment limit" that forces the algorithm to terminate and output intermediate pathway fragment results *E*(*K*^*i*^) at the first iteration *i *that this limit is met. In our implementation, we also apply heuristic "local greedy" optimization strategy that dynamically rearranges the rows of *S *at the end of each iteration with the goal of minimizing computation at the next iteration [15]. Specifically, at each iteration *i*, we rearrange rows *i *+ 1, ..., *m *of *S *to minimize the number of ray pairs in *E*(*K*^*i*^) that lie on opposite sides of hyperplane *S*_*i*+1_*v *= 0.

### Minimal hitting set algorithm

We compute the H of all minimal hitting sets of cardinality *k *or less of a collection *E *of rays (i.e. pathway fragments) using *MinHit *(Table 3), which is a modification of the Berge algorithm [26]. In *MinHit*, a collection of minimal hitting sets for the collection of rays *E *is built through an iterative traversal through *E*. The initial collection contains a single set: the empty set. At each iteration *q*, a new collection is built by augmenting sets in the old collection that fail to intersect the positive components in the current ray. Each non-intersecting set is augmented by combining it with an element corresponding to a single positive component of the current ray. The latter is done for positive component of the current ray and every non-intersecting set in the old collection. After each set is augmented in this manner, the new collection is scanned to eliminate sets that are non-minimal (i.e. for which there exists a subset in the collection). The resulting collection can be easily shown to form a minimal hitting set collection for the rays 1...*q *in collection *E *that have already been processed. Upon iterating through the entire collection *E*, the output H is a minimal hitting set for *E*.

**Table 3:** 

H = *MinHit*(*E*, *k*, *R*)
1: // *Arguments:*
2: // *E *= {*r*^1^, ..., *r*^|*E*|^}, a collection of rays in ℝ^*n*^.
3: // (e.g. *j*-containing pathway fragments)
4: // *k*, a cardinality cutoff (infinite by default).
5: // *R*, a subset of *N *to which the search
6: // for minimal hitting sets can be limited
7: // (= *N *by default)
8: // *Output:*
9: // H, a collection of minimal hitting sets for *E*
10: // (with cardinality *k *or less, limited to subsets of *R*)
11: H = {∅}
12: **for all ***r *∈ *E ***do**
13: $H_{temp}$ = {}
14: //Augment non-intersecting sets in H to
15: //"hit" nonzero components of ray *r *of *E*
16: **for all ***H *∈ H**do**
17: //If the *H *does not intersect the nonzero
18: //components of *r*, then augment *H*
19: //and add to $H_{temp}$
20: **if ***H *⋂ *NZ*(*r*) = ∅ **then**
21: **for all ***i *∈ *R *for which *r*_*i *_≠ 0 **do**
22: add *H *∪ {*i*} to $H_{temp}$
23: **end for**
24: **else**
25: add *H *to $H_{temp}$
26: **end if**
27: **end for**
28: //Populate $H_{new}$ with minimal, non-duplicate, sets
29: //from $H_{temp}$ that meet the cardinality threshold
30: H = {}
31: **for all ***H *∈ $H_{temp}$**do**
32: **if ***H *∉ $H_{new}$**and **${∄}_{\hat{H}\in H_{temp}}$ | $\hat{H}$ ⊂ *H ***and **|*H*| ≤ *k ***then**
33: add *H *to $H_{new}$
34: **end if**
35: **end for**
36: H = $H_{new}$
37: **end for**
38: return H

Additional arguments to *MinHit *are a cardinality limitation *k *and a subset of indices of interest *R *to which minimal hitting sets can be restricted. The cardinality limitation is implemented by discarding all sets with cardinality greater than *k *at each iteration. The subset limitation is implemented by only considering elements in *R *at each augmentation step.

### Reducing non-minimal cut sets

We apply an LP based procedure called *Reduce *(Table 4) to reduce non-minimal cut sets (represented by collection H) for an objective reaction set *J *to minimality. For each cut set *H *in H, subsets of cardinality |*H*| - 1 are tested to see if they are cut sets for *J*. If none is found, then this cut set *H *is shown to be minimal. Otherwise, the newly found cut sets are added to a cardinality-sorted collection *ToDo *and later tested for minimality in the same way. When a given subset of *H *is shown to be minimal or non-minimal, all of its super-sets are removed from *ToDo*. The final output of *Reduce *is an LP verified collection of MCS for the objective set *J*.

**Table 4:** 

*MCS *= *Reduce*(H, *J*, *S*)
1: // *Argument:*
2: // H, a collection of cut sets for objective reaction
3: // set *J *⊂ *N *in system *S *∈ ℝ^*m *× *n*^
4: // *Output:*
5: // *MCS*, a collection of MCS for *J*
6:
7: *MCS*, *Done *= empty collection
8: **for all **sets *H *in collection H**do**
9: *ToDo *= {*H*};
10: **while ***ToDo *is not empty **do**
11: $\bar{H}$ = a lowest cardinality set from *ToDo*
12: //Apply LP based test to all
13: //immediate subsets of $\bar{H}$ to determine
14: //which cut *at least one *flux in objective set *J*
15: D = collection of size |$\bar{H}$| - 1 subsets of $\bar{H}$ that are also cut sets for objective set *J *in *S*
16: add $\bar{H}$ to collection *Done*
17: **if **D is empty **then**
18: add $\bar{H}$ to collection *MCS*
19: **else if **D contains one set **then**
20: add sole set in D to *MCS *and *Done*
21: **else**
22: add sets in D to *ToDo*
23: **end if**
24: Remove from *ToDo *super sets of any set in collection *Done*
25: **end while**
26: **end for**
27: return *MCS*

### Minimal cut set computation in *E. coli *iJR904

We use the computational framework developed in this paper to compute MCS for biomass production in the *E. coli *iJR904 genome scale model [8], which has 761 metabolites involved in 931 reversible and irreversible chemical reactions. We convert each reversible reactions into two irreversible reactions and supplement the network with 143 extracellular nutrient FLuxes, 5 intracellular nutrient fluxes representing supply of cofactors and carrier proteins from outside of small molecular metabolism, and 761 species sink fluxes for each species in the system. This yields a stoichiometry matrix *S *(Equation 2) of dimension 761 × 2085.

Reactions in *S *represent the inflow, outflow, and interconversion of small-molecule chemical species in an *E. coli *cell grown in a rich nutrient media. Each species "sink" reaction represents its growth mediated dilution and macromolecular consumption. Of the 761 species, 49 correspond to "biomass components" that are considered to be essential substrates for survival and growth in the original Reed *et al *specification [8]. In this model, knock out of biomass production (and thus growth and survival) corresponds to "cutting" flux through *at least one *of the sinks corresponding to an essential biomass component.

We then applied the batch procedure *NetKO *(Table 5) to the *E. coli *stoichiometry matrix *S *compute MCS with cardinality limit *k *= 10 for the collection *J *of biomass sinks, limited to subsets of non-sink reactions *R *⊂ *N*. Briefly, *NetKO *first applies the tableau algorithm to generate the set *E *of pathway fragments using the "local greedy" optimization strategy outlined by Bell and Palsson [15]. The tableau algorithm terminates at an iteration at which the number of pathway fragments exceeds the limit *PFL*. For this implementation, we set *PFL *= 150,000 according to what we found to be feasible given our current computing resources. *NetKO *then calls *MinHit *for each biomass sink *j *∈ *J *to generate cut sets of cardinality *k *from the collection of *j*-containing pathway fragments *E*^*j*^, limited to the subset *R*. The procedure then removes non-minimal cut sets from the collection H of pooled results through a pairwise set comparison and applies *Reduce *to generate the final collection of MCS.

**Table 5:** 

*MCS *= *NetKO*(*S*, *J*, *k*, *R*, *PFL*)
1: // *Arguments:*
2: // *S*, stoichiometry matrix
3: // *J *⊂ *N*, a set of objective reactions
4: // *k*, cardinality limit for MCS
5: // *R*, reactions from which MCS should be chosen
6: // *PFL*, pathway fragment limit for tableau algorithm
7: // *Output:*
8: // *MCS*, a collection of MCS for *J*
9: *E *= *tableau*(*S, PFL*)
10: H = {}
11: **for all ***j *∈ *J ***do**
12: *E*^*j *^= subset of *j*-containing pathway fragments in *E*
13: $H^{j}$ = *MinHit*(*E*^*j*^, *k*, *R*);
14: H = H ∪ $H^{j}$
15: **end for**
16: remove sets *H *∈ H from H for which there exists a subset in H
17: *MCS *= *Reduce*(H, *J*, *S*)
18: return *MCS*

Application of the *NetKO *procedure *E. coli *IJR904 network in rich media yielded 11,706 MCS for biomass production. Analyzing the performance of individual steps in *NetKO*: the tableau algorithm generated 164,093 pathway fragments following execution of 708 of 761 iterations of the tableau algorithm during about 2 hours of execution. Application of the *MinHit *algorithm (Table 3) to this pathway fragment collection yielded 71,230 cut sets of cardinality *k *≤ 10 that disable flux through one or more of the 49 biomass component sinks. This computational step required just over 2 minutes to execute. Of the cut sets produced by *MinHit*, 59,279 could be shown to be non-minimal via pair wise comparison to other cut sets in the collection. Application of the LP-based *Reduce *procedure to the remaining 11951 cut sets yielded 11,706 MCS for biomass production. Though very few cut sets in the input to *Reduce *were non-minimal, this step was the most expensive computationally, requiring 113,989 linear programs over about 12 hours of computation.

We also computed MCS using a brute-force LP knockout analysis by determining the feasibility of flux through the collection of biomass sinks *J *for each single and double reaction knockout in the network. For the analysis of double knockouts, we only considered reaction knockout pairs that failed to disable biomass production as single reaction knockouts. When performing brute-force LP analysis, we treated knockout reaction pairs corresponding to the same reversible reaction as single knockouts.

All algorithms were written and executed within the MATLAB programming environment on a Pentium 2.0 GhZ PC with 2 GB of RAM. We used the semidefinite programming package SeDuMi for MATLAB to implement the linear flux feasibility analysis used in *Reduce *and the brute-force LP approach [27]. In this flux feasibility analysis, we evaluate whether a reaction set *C *is a cut set for objective set *J *by checking the feasibility of {*v *∈ *K*|*v*_*C *_= 0, *v*_*j *_≠ 0} for each objective *j *∈ *J *through analysis of primal and dual feasible points in a sequence of linear programs. In the vast majority of knockouts (and all knockouts examined in this study), this procedure determines the feasibility of fluxes in *J *through the execution of a single linear program.

## Authors' contributions

MI formulated the theory, implemented the algorithm, designed the study, conducted the analysis, and drafted the manuscript. CB helped to formulate the theory and draft the manuscript. All authors read and approved the final manuscript.

## Supplementary Material

Additional file 1 Supplementary Data. The data provided lists the reaction labels corresponding to each minimal cut set discovered by the *NetKO *algorithm for *E. coli *biomass production in rich media. Click here for file
